# Understanding the role of 5d electrons in ferromagnetism and spin-based transport properties of K_2_W(Cl/Br)_6_ for spintronics and thermoelectric applications

**DOI:** 10.1039/d2ra01841a

**Published:** 2022-10-31

**Authors:** Mukaddar Sk, Saurabh Ghosh

**Affiliations:** Department of Physics and Nanotechnology, SRM Institute of Science and Technology Kattankulathur 603 203 Tamil Nadu India mukaddarsk05@gmail.com saurabhghosh2802@gmail.com

## Abstract

In this article, we have systematically investigated the structural, electronic, magnetic, and spin-based thermoelectric properties of K_2_W(Cl/Br)_6_ by first-principles calculation. The obtained negative formation energy confirmed the thermodynamic stability of K_2_W(Cl/Br)_6_, while the tolerance factor calculation showed their cubic phase stability. In addition, we have estimated the elastic constants which confirmed the mechanical stability of K_2_W(Cl/Br)_6_. Further, the spin-polarized band structure and density of states calculations revealed the half-metallic nature with high Curie temperature (*T*_c_) values of 613 K and 597 K for K_2_WCl_6_ and K_2_WBr_6_, respectively. Moreover, we have studied the temperature variation of thermoelectric properties such as *k*_l_, *σ*, *k*_e_, *S*, PF, and *ZT*. Such results showed that higher *ZT* values for spin-down channels are obtained from ultra-low *k*_e_, and high PF. Therefore, K_2_W(Cl/Br)_6_ are viable thermoelectric and spintronic materials.

## Abstract 2

The DFT calculation showed the half-metallic nature of K_2_WCl_6_ and K_2_WBr_6_ with their high *T*_c_. Furthermore, the thermoelectric calculation showed that higher ZT values of K_2_WCl_6_ and K_2_WBr_6_ originated from ultra-low ke and high PF.

## Graphical abstract

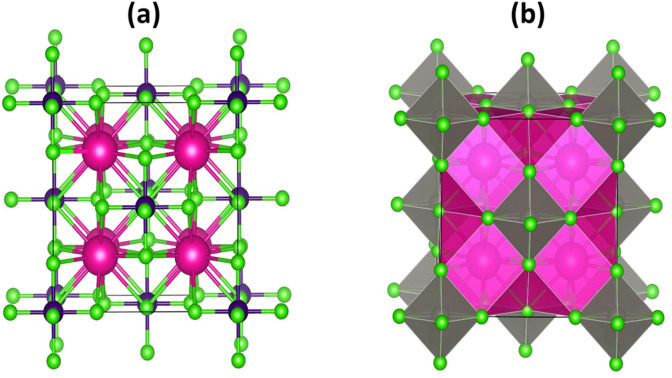

## 1. Introduction

The advancement of spintronics and quantum computing technology has boosted the memory storage speed with multifunctional characteristics. This is a developing field which reduces magnetic chip size and enhances the memory speed by utilizing the electron spin and its charge.^1,2^ The advanced spintronic technology also has novel achievements in non-volatile magnetic random-access memory.^3,4^ These advancements can also be applied in electronic devices due to their low cost, faster data speed, and less power consumption.^5–7^ In addition, the recent quantum technology takes advantage of electronic spin states in the digital display rather than the charge states of a typical electronic operation. The development of giant magnetic resistance (GMR) in 1998 boosted this technology,^8^ where the electron spin can create an enormous difference in the resistance of alternative magnetic layers in the presence of external applied magnetic fields. Thus, the magnetic response of electronic charge and spin facilitate the low-powered, high-speed, non-volatile, and nano-size memory.^9,10^

The present scientific achievements of spintronics technology gave rise to the improvement of magneto-resistive random-access memory (MRAM), magnetic sensors, magnetic valves, read heads of magnetic hard drives, and giant magneto-resistive effect (GMR). Materials exhibiting the high spin polarization (SP) are suitable candidates for spintronics technology. Rather, for the half-metallic ferromagnetism (HMF) materials, one channel is metallic and the other one is insulating, which produces 100% SP, demonstrating that they are spintronics materials.^11,12^ The first HMF was observed by de Groot *et al.* in Heusler alloy PtMnSb and NiMnSb in 1983.^13^ After this, HMF was observed in many types of materials such as diluted magnetic semiconductors, perovskites, spinel chalcogenides, and double perovskites.^14,15^ But, the major confront of spintronic materials are their phase instability at elevated temperature and clustering of magnetic ions greatly influences the functionality of spin. In later research, the phase instability issue was resolved at higher temperature, but the problem of clustering remains to be addressed. Nonetheless, a lot of transition-metal doped alloys were formed at room temperature, still the issue of clustering limits their applications.^16^ To solve the problem of spin segregation, numerous procedures were adopted, where the doping of nonmagnetic elements into alloys is prominent. The FM behaviour has been reported in Be_1−*x*_C_*x*_S,^17^ Sn_1−*x*_Mg_*x*_O_2_,^18^*etc.* But these materials are expensive and have complicated production procurements.

Recently, Halide based double perovskites with chemical formula X_2_YZ_6_ (X = Cs, Rb, K Y = Os, Nb, Ta, W; Z = Cl, Br, I) have attracted considerable attention for spintronics applications due to their low cost, high Curie temperature (*T*_c_), and good stability.^19–23^ In X_2_YZ_6_, the d-orbital electrons of Y atom contributes significantly to the magnetic moment. Furthermore, it has been noted that the halide based double perovskites have ferromagnetism at high Curie temperature. However, there is a lack of detailed description on the physical properties of X_2_YZ_6_. In this article, we have taken K_2_W(Cl/Br)_6_ compounds to investigate their magnetic and spin-based transport properties by first-principles calculation. These K_2_W(Cl/Br)_6_ compounds have been prepared by Xu *et al.* and Epperson *et al.* from a stoichiometric mixture of K(Cl/Br) and W(Cl/Br)_4_ and they have observed the cubic phase stability from the X-ray diffraction patterns.^24,25^ However, existing literatures about these compounds are only limited to their structural studies. To the best of our knowledge, there is no detailed report available on magnetic and spin-based transport properties of K_2_W(Cl/Br)_6_. Our spin-polarized band structure and density of states calculations revealed the presence of half-metallic character in these materials with high Curie temperature. Thus, K_2_W(Cl/Br)_6_ compounds are emerging spintronics materials.

Besides spintronics applications, the lead-free halide based double perovskites have attracted considerable attention for photovoltaic and thermoelectric technology. A good photovoltaic material should have high optical absorption coefficient and conductivity, with low reflectivity. On the other hand, a material is efficient for thermoelectric technology, if it has high Seebeck coefficient (*S*), good electrical conductivity (*σ*), and low thermal conductivity (*k*).^26^ Many lead-free halide based double perovskites are reported to have suitable photovoltaic and thermoelectric properties. For instance, Haq *et al.* predicted that Rb_2_XGaBr_6_ (X = Na, K) are promising photovoltaic and thermoelectric materials due to their optimum optical absorption coefficient and large *ZT* values, respectively.^52^ Later, Iqbal *et al.* have shown the emerging photovoltaic and thermoelectric properties of Rb_2_AlInX_6_ (X = Cl, Br, I) due to their narrow band gap.^53^ Also, Nawaz *et al.* reported that Rb_2_YInX_6_ (*X* = Cl, Br, I) are thermodynamically stable and they are suitable for photovoltaic and thermoelectric technology.^54^ Instead of low thermal conductivity arising from the occupation of cations in the octahedral structure, it is very surprising that these halide based double perovskites are mainly studied for photovoltaic purposes. Only very few experimental studies were performed to investigate their thermoelectric properties and interestingly the research in the thermoelectric response is now growing.^27^ In this article, we have carried out the spin-based thermoelectric properties of K_2_W(Cl/Br)_6_. We have computed the temperature variation of *k*_l_, *σ*, *k*_e_, *S*, PF, and *ZT*. The higher *ZT* values for spin-down channels have resulted from ultra-low *k*_e_, and high PF. Thus, K_2_W(Cl/Br)_6_ are potential thermoelectric and spintronic materials.

## 2. Computational details

The electronic structure, magnetic properties and transport properties of K_2_W(Cl/Br)_6_ were investigated by using Wien2k^28^ and BoltzTraP code.^29^ We have employed PBEsol approximation to optimize the crystal structure in FM and AFM states.^30^ However, PBEsol approximation underestimates the electronic bandgap on which magnetic and transport properties are dependent. Therefore, we have employed TB-mBJ formalism,^31^ which can accurately predict the bandgap. In addition, due to the presence of heavy elements, SOC coupling is significant, that was added with TB-mBJ. The energy cut-off for geometry optimization was selected to be 520 eV. On the other hand, the average forces per ions were optimized to 0.002 eV Å^−1^. Furthermore, we have considered a *k* mesh of 12 × 12 × 12 for electronic calculation. The product of maximum wave vector and muffin tin radii is kept as *R*_MT_ × *K*_max_ = 8, along with angular momentum vector *l* = 10 and Gaussian factor = 10. The level convergence is achieved to be 10^−5^ Ry self consistently by using the above-mentioned inputs. For transport properties calculation, we have considered a dense *k*-points of 150 000.

## 3. Results and discussion

### 3.1 Structural and mechanical stabilities

**Fig. 1:**
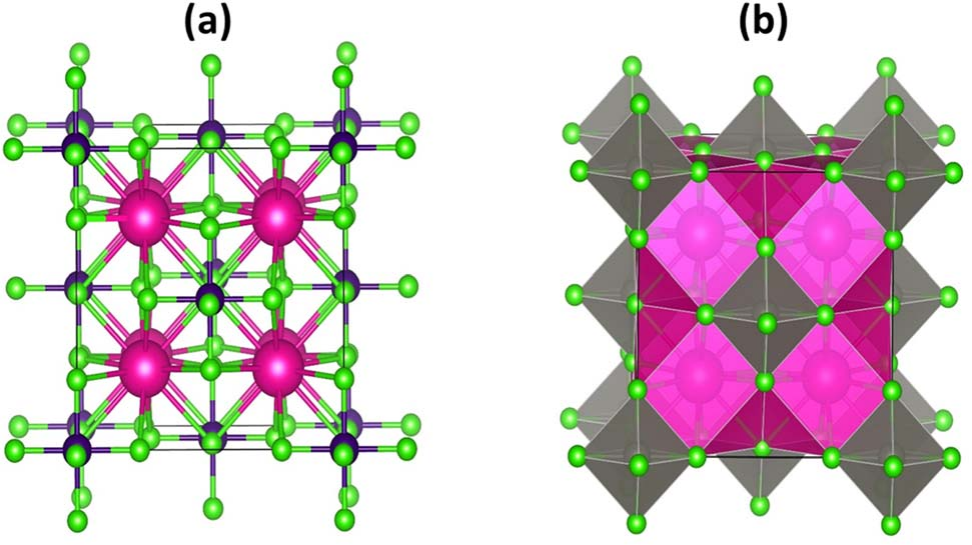
The crystal structure (a) atomic and (b) polyhedral forms of K_2_W(Cl/Br)_6_ with magenta, blue and green colors represent the K, W, and (Cl/Br) atoms respectively.

The halide based double perovskites K_2_W(Cl/Br)_6_ have a cubic phase with space group *Fm*3̄*m* (225).^32^ The perspective view of K_2_W(Cl/Br)_6_ is shown in Fig. 1. The vacancies between the octahedra W(Cl/Br)_6_ are occupied by K atoms whereas each octahedron is separated by the other octahedra through the 12-fold coordination system of (Cl/Br). In this structure, each K atom is surrounded by 12 (Cl/Br) atoms, whereas every W atom is coordinated with 6 (Cl/Br) atoms. Moreover, each W(Cl/Br)_6_ is located at the corner and face center of the cubic system. The K, W, and (Cl/Br) atoms in the unit cells of both systems are positioned at (0.25, 0.25, 0.25) (0, 0, 0) and (*x*, 0, 0), respectively. The atomic positions in the structure are corrected by minimizing strain throughout the optimization process.

**Fig. 2:**
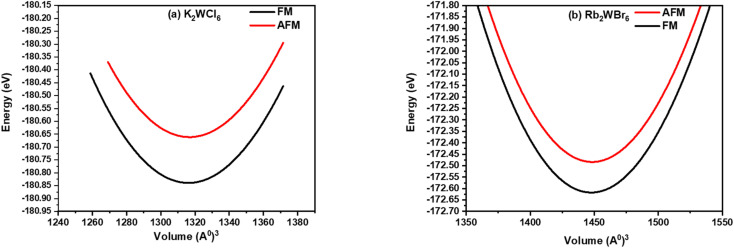
Volume optimization plot of (a) K_2_WCl_6_ and (b) K_2_WBr_6_ in FM (black line) and AFM (red line) calculated through PBEsol approximation.

The optimized energy *versus* volume plot is shown in Fig. 2(a and b). It is noticeable from Fig. 2(a and b), that K_2_W(Cl/Br)_6_ compounds have positive energy difference between FM and AFM states indicates that the FM is more preferable because of more energy release in this process.

Curie temperature (*T*_c_) of K_2_W(Cl/Br)_6_ are predicted through the Heisenberg classical model. e., *T*_c_ = 2Δ*E*/3*xK*_B_, where x is the contribution of W atom and Δ*E* is the energy difference between ferromagnetic and antiferromagnetic ground states, *i.e.* Δ*E* = *E*_AFM_ − *E*_FM_.^33,34^ The computed *T*_c_ values are 613 K and 597 K for K_2_WCl_6_ and K_2_WBr_6_, respectively. The high *T*_c_ values make these compounds suitable for spintronic applications.

The cubic phase stability of K_2_W(Cl/Br)_6_ are investigated from Goldsmith tolerance fact or calculation as follows.1
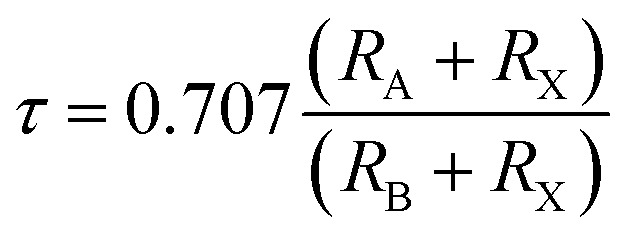


It is worthy to mention here that, the stable cubic phase should have *τ* value in the range of 0.9–1.02.^35^Table 1 clearly shows that *τ* values of K_2_W(Cl/Br)_6_ are in this range demonstrating their cubic phase stability.

**Table 1:** The obtained lattice parameter (*a*), tolerance factor (*τ*), octahedral factor (*μ*), and formation energy (Δ*H*_f_) of K_2_W(Cl/Br)_6_

Compound	a (Å)	*τ*	*μ*	Δ*H*_f_ (eV)
K_2_WCl_6_	10.71	0.98	0.45	−31.12
K_2_WBr_6_	10.75	0.98	0.44	−27.12

To investigate the synthetic possibility of K_2_W(Cl/Br)_6_, we have calculated the enthalpy of formation by using the following equation

2 Δ*H*_f_ = *E*_total_{K_2_W(Cl/Br)_6_)} − 2*E*^bulk^_K_ − *E*^bulk^_W_ − 6*E*^bulk^_(Cl/Br)_

where, *E*_total_{K_2_W(Cl/Br)_6_}, *E*^bulk^_K_, *E*^bulk^_W_ and *E*^bulk^_(Cl/Br)_ represent the ground state energy of K_2_WX_6_, K, W, and (Cl/Br) in their bulk form. The predicted values of Δ*H*_f_ are −31.12 eV and −27.12 eV for K_2_WCl_6_ and K_2_WBr_6_, respectively. This negative Δ*H*_f_ revealed the thermodynamic stability of K_2_W(Cl/Br)_6_.

The mechanical stability was studied from elastic constant values (*C*_ij_), obtained by using the Cubic Elastic package.^56^ It is observed from Table 2 that the values of the various components of *C*_ij_ obeyed the Born criteria of mechanical stability, *i.e. C*_11_ − *C*_12_ > 0, *C*_44_ > 0, *C*_11_ + 2*C*_12_ > 0, *C*_12_ < *B* < *C*_11_.^37^ Furthermore, we have estimated the Cauchy's pressure (CP = *C*_12_–*C*_44_). The positive value of CP demonstrated the ductile properties of K_2_W(Cl/Br)_6_. The overall elastic study confirmed that K_2_W(Cl/Br)_6_ are mechanically stable.^36^

**Table 2:** The calculated bulk modulus (*B*), elastic constants (*C*_ij_), and cauchy pressure (CP) of K_2_W(Cl/Br)_6_

Compound	*B* (GPa)	*C*_11_	*C*_12_	*C*_44_	CP
K_2_WCl_6_	41.16	78.19	21.17	18.72	2.45
K_2_WBr_6_	39.33	74.89	20.76	18.41	2.35

### 3.2 Magnetic properties

**Fig. 3:**
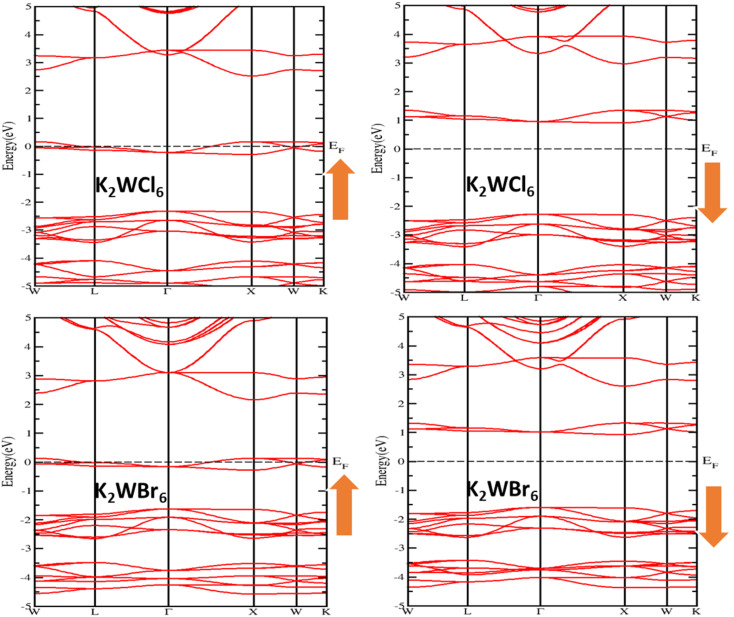
The spin-polarized band structure of K_2_W(Cl/Br)_6_ calculated through mBJ + SOC.

The half-metallic ferromagnetic material having high spin polarization (*P*) is essential for spintronic applications. The spin polarization (*P*) can be obtained as3
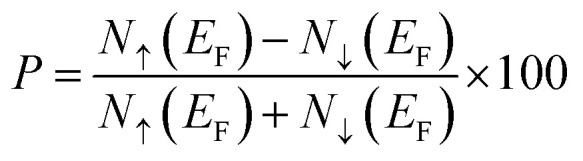
where (*N*_↑_) and (*N*_↓_) represent the density of states of up and down the channels at Fermi level (*E*_F_). The spin-polarized band structure is shown in Fig. 3. It is noticeable from Fig. 3 that, the quantum state of the spin-up channel overlapped with Fermi level (*E*_F_), which demonstrates the metallic nature. However, the spin-down channel shows the insulating nature because of the existence of finite separation between valence band maxima (VBM) and conduction band minima (CBM). Therefore, the combined spin up and spin down showed the half-metallic ferromagnetism nature with 100% spin polarization (*P* = 1). The detailed investigation revealed that the valence band maxima (VBM) and conduction band minima (CBM) of spin-down channels are located at the same *k*-point for both compounds, which indicates the direct band gap properties. We have obtained the band gap of 3.01 eV and 2.72 eV for K_2_WCl_6_ and K_2_WBr_6_, respectively in the spin-down channel. Also, the estimated total magnetic moment is calculated to be 2*μ*_B_ for both compounds. The integer value of total magnetic moment implies that K_2_W(Cl/Br)_6_ are half metallic ferromagnets. Thus, K_2_W(Cl/Br)_6_ are emerging spintronics materials.

**Fig. 4:**
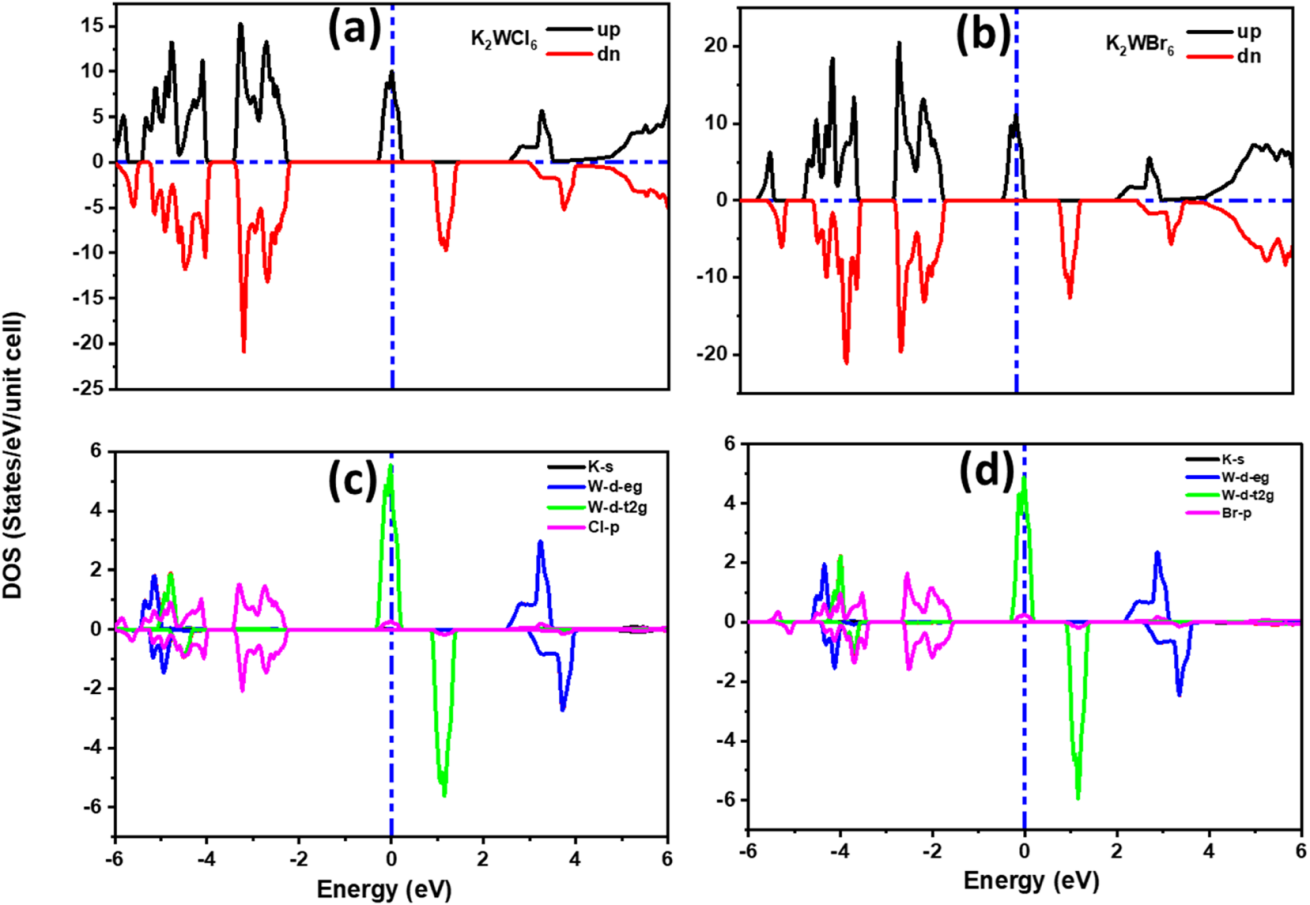
The spin polarized (a and b) total DOS, (c and d) partial DOS of K_2_W(Cl/Br)_6_ obtained with mBJ + SOC.

For the detailed investigation of band structure results, we have further calculated the total and partial density of states of K_2_W(Cl/Br)_6_ as shown in Fig. 4(a–d). It is noticeable from Fig. 4(a and b) that the total DOS in the up spin showed the metallic behavior and the down spin presented the insulating behavior. Therefore, K_2_W(Cl/Br)_6_ are half-metallic ferromagnetism (HMF) in nature. To investigate the origin of half-metallic ferromagnetism (HMF) in K_2_W(Cl/Br)_6_, we have further investigated the partial density of states (PDOS) which is shown in Fig. 4(c and d). It is evident from Fig. 4(c and d) that the d-t_2g_ states of W atoms are responsible for introducing HMF in K_2_W(Cl/Br)_6_. In the spin-up case, d-t_2g_ states of W atoms overlapped with Fermi level (*E*_F_), which produces the metallic nature in K_2_W(Cl/Br)_6_. In the spin-down case, d-t_2g_ states move deep into the conduction band and thereby leaving a finite separation between the valence band and conduction band and therefore create insulating nature in K_2_W(Cl/Br)_6_.

**Fig. 5:**
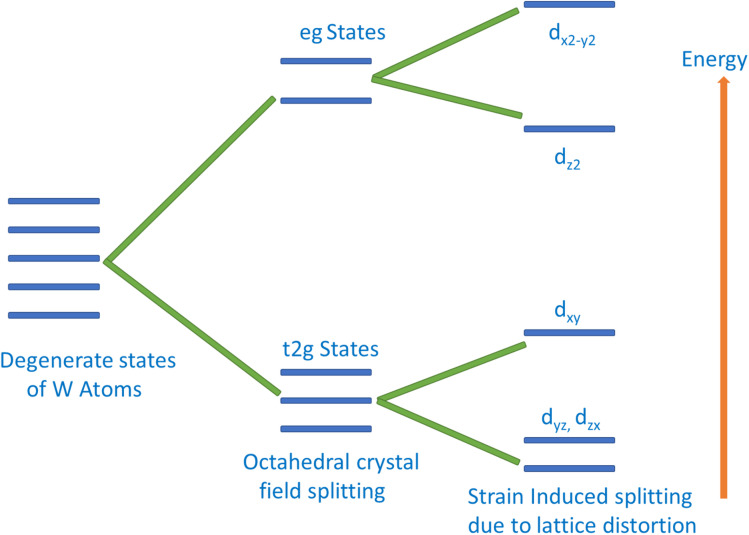
Schematic representation of crystal-field splitting of W-5d orbital.

The magnetic behavior of K_2_W(Cl/Br)_6_ has also been addressed by an exchange mechanism. The crystal field due to the Coulomb interaction between W and (Cl/Br) atoms split the 5d states of W into t_2g_ and e_g_. The splitting of W-5d states is schematically shown in Fig. 5. It is clearly noticeable from Fig. 5 that the t_2g_ is triply degenerate (d_*xy*_, d_*yz*_, d_*zx*_), which has lower energy than the double degenerate e_g_ (d_*z*^2^_, d_*x*^2^_ − _*y*^2^_) states. The splitting of e_g_ states results in the upward and downward shifting of d_*x*^2^_ − _*y*^2^_ and d_*z*^2^_, respectively. At the same time, splitting of t_2g_ state results in the increase of d_*xy*_ state energy and decrease of d_*yz*_ and d_*zx*_ states energies. The major magnetic response originated from the t_2g_ state of the W atom. This state is hybridized with the p state of (Cl/Br) *via* double exchange mechanism. Thus, we conclude that the double exchange mechanism between W atoms *via* (Cl/Br) atom is responsible for introducing HMF in K_2_W(Cl/Br)_6_.

### 3.3 Thermoelectric properties

Solid-state thermoelectric materials are gaining considerable attraction as novel materials for converting thermal to electrical energy.^38–42^ Many mechanical and electronic devices release a huge amount of heat as waste. To take the advantage of wasted heat, efficient thermoelectric materials are needed which can directly convert the wasted heat into electricity. The thermoelectric performance of a material is given by a dimensionless parameter known as a figure of merit (*ZT*) given by; 
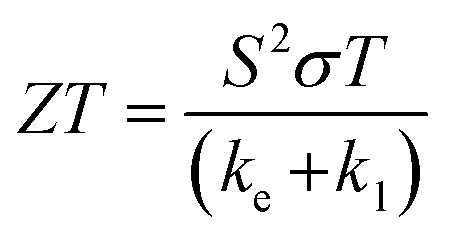
, where *σ*, *S*, *T*, *k*_e_, and *k*_l_ represent the electrical conductivity, Seebeck coefficient, temperature, electrical thermal conductivity, and lattice thermal conductivity, respectively. The material with a large *ZT* value is considered to be a potential candidate for thermoelectric technology. But, obtaining a large *ZT* value is challenging due to the strong correlation of these physical properties. According to a recent report, magnetic interaction is one of the suitable approaches to enhance *ZT*.^43–47^ The interaction of charge carriers with local magnetic moments, can enhance the carrier effective mass (m*), consequently thermoelectric power (*S*^2^*σ*) and corresponding figure of merit (*ZT*). It is worthy to mention here that such magnetic interaction is present in K_2_W(Cl/Br)_6_. Due to the presence of magnetic interaction in K_2_W(Cl/Br)_6_, it is expected that these systems will have large *ZT* values. However, there are no detailed studies on thermoelectric properties of K_2_W(Cl/Br)_6_.

In this article, we have calculated the temperature variation of thermoelectric properties in K_2_W(Cl/Br)_6_. For this, we have employed BoltzTrap code.^24^ During all calculations, the relaxation time (*τ*) is fixed by BoltzTraP code as *τ* = 10^−14^ s. We have separately calculated the thermoelectric parameters for spin-up and spindown configurations.

**Fig. 6:**
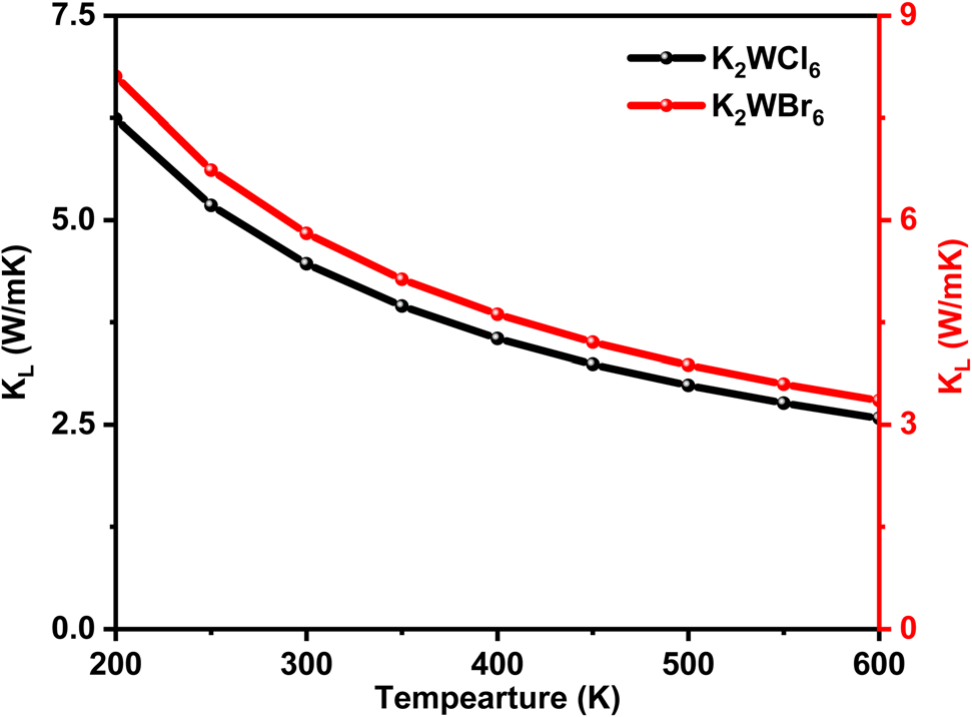
The temperature-dependent lattice thermal conductivity (*K*_l_) of K_2_W(Cl/Br)_6_ calculated through the Slack equation.

For thermoelectric calculation, the temperature was varied from 200 K to 600 K. The total thermal conductivity (*k*) is the sum of electronic (*k*_e_) and lattice (*k*_l_) thermal conductivities. A good thermoelectric material should have low *k*. The temperature variation of lattice thermal conductivity (*k*_l_) was calculated by using the Slack equation.^55^ The temperature variation of *k*_l_ is shown in Fig. 6. It can be seen from Fig. 6 that *k*_l_ has an inverse relationship with temperature, which is a typical feature of half-metallic ferromagnetic materials.^48^ It is worthy to mention here that K_2_W(Cl/Br)_6_ compounds have low *k*_l_ values.

The amount of charge flow per unit time inside of a compound can be understood from its electrical conductivity (*σ*). The materials are categorized into insulator, semiconductor, and metal-based on their ability of charge flow.^49^ A good thermoelectric material should have a large *σ* value.^50^ The temperature variation of *σ* is calculated for K_2_W(Cl/Br)_6_ as shown in Fig. (a) and Fig. 8(a), respectively. The detailed analyses revealed that the *σ* value for spin-up configurations in K_2_W(Cl/Br)_6_ decreases with temperature until achieving the lowest value of 1.97 × 10^5^ Ω^−1^ m^−1^ (K_2_WCl_6_) and 2.25 × 10^5^ Ω^−1^ m^−1^ (K_2_WBr_6_) at 600 K. On the other hand, the values of *σ* for spin-up states in K_2_W(Cl/Br)_6_ are almost constant in the entire temperature up to 400 K. However, there was a gradual increase of *σ* is observed above 400 K. This trend of *σ* for up and down channels is typical feature in HMF.^51^ The detailed investigation reveals that spin-up channel *σ* is dominant in both cases.

**Fig. 7:**
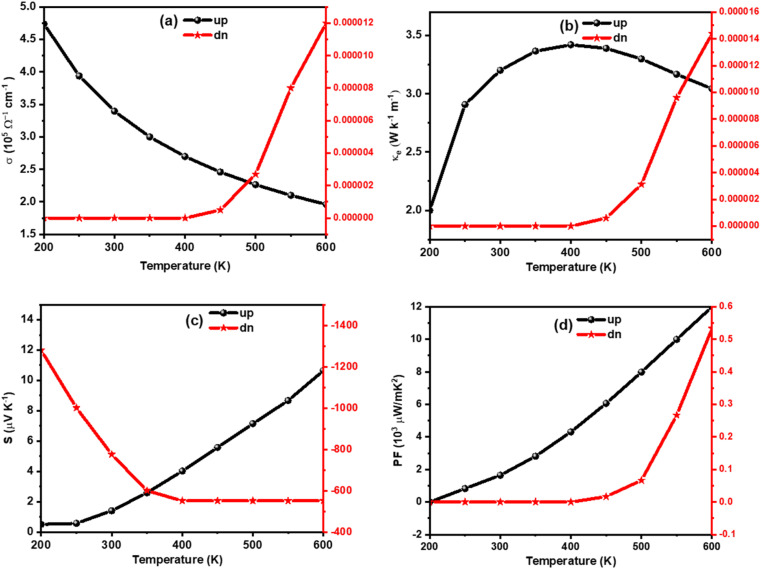
Calculated (a) *σ*, (b) *k*, (c) *S*, and (d) PF of K_2_WCl_6_ as a function of temperature.

**Fig. 8:**
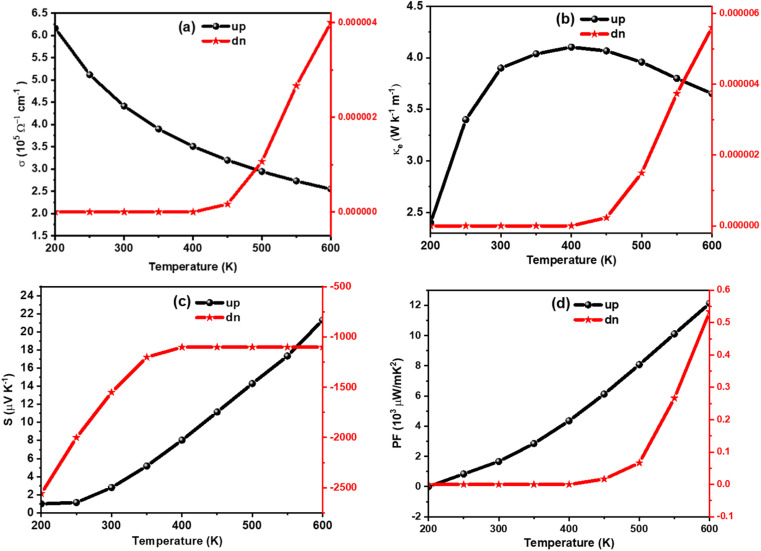
Calculated (a) *σ*, (b) *k*, (c) *S*, and (d) PF of K_2_WBr_6_ as a function of temperature.

We have also examined the temperature variation of electronic thermal conductivity (*k*_e_) as shown in Fig. 7(b) and 8(b) for K_2_WCl_6_ and K_2_WBr_6_, respectively. It is noticeable that *k*_e_ for spin-up configuration has a direct relation with temperature up to a certain limit where *k*_e_ increases to 3.41 W K^−1^ m^−1^ and 4.1 2 W K^−1^ m^−1^ respectively for K_2_WCl_6_ and K_2_WBr_6_, at 400 K. Above this temperature, *k*_e_ decreases gradually for both systems. On the other hand, the values *k*_e_ in spin-down states of K_2_W(Cl/Br)_6_ are 0.0 in the entire temperature up to 400 K. Above this temperature, *k*_e_ increases abruptly in both cases. It is worthy to mention here that *k*_e_ values for spin-down channels are very less compared to spin-up configuration.

The Seebeck coefficient (*S*) plays an important role to describe the thermoelectric performance. The *S* is defined by the ratio of a voltage difference to that of a temperature difference. It can also show the capability of a material to generate the thermo-electromotive force from a given temperature gradient. The *S* can be calculated by the following relation4
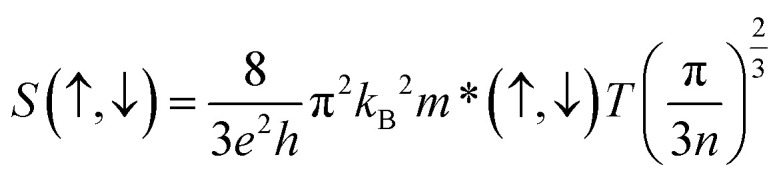
where *h*, *k*_B_, *e*, m*(↑,↓), *T*, and *n* represent the Planck constant, Boltzmann constant, electronic charge, spin-dependent carrier effective mass, absolute temperature, and carrier concentration respectively. A good thermoelectric material should have a large Seebeck coefficient. Fig. 7(c) and 8(c) presented the Seebeck coefficients for both compounds in both spin configurations as a function of temperature. It is noticeable from Fig. 7(c) and Fig. 8(c) that the obtained values of Seebeck coefficients are positive in the entire temperature range of spin up channels, demonstrating the presence of p-type charge carriers (hole), whereas negative values Seebeck coefficients for a spin up channel suggest the presence n-type charge carriers (electron). The absolute value of S for spin up configuration in K_2_WCl_6_ (K_2_WBr_6_) increases from 0.67 μV K^−1^ (1.01 μV K^−1^) at 200 K linearly up to 600 K. This increasing trend in up channel is due to its metallic nature. For metal, there is huge number of free electrons and hence applying a temperature gradient should lead to diffusion of more charge carriers towards cold end. As a result, the potential difference between two ends will increase and therefore *S* will increase. In the spin-down channel, there is an abrupt decrease of *S* in K_2_WCl_6_ (K_2_WBr_6_) from a very high value of 1267 μV K^−1^ (1397 μV K^−1^) at 200 K beyond this temperature *S* decreases gradually. This, decrease trend in spin-down channel can be described from eqn (4). The increasing of temperature can enhance the carrier concentration and thereby decreasing the value of *S*. The detailed investigation depicts that spin down channel Seebeck coefficient is dominant in both cases.

In addition, we have calculated the thermoelectric power factor (PF) as shown in Fig. 7(d) and Fig. 8(d). It is noticeable that the PF of spin-up configuration in K_2_WCl_6_ (K_2_WBr_6_) increases from 0.02 × 10^9^ W mK^−1^ (0.01 × 10^9^ W mK^−1^) at 200 K linearly up to 600 K. On the other hand, the PF of spin-down state is found to be 0.0 μW mK^−2^ for both systems in the entire temperature up to 400 K. Beyond this temperature, the PF increases gradually.

**Fig. 9:**
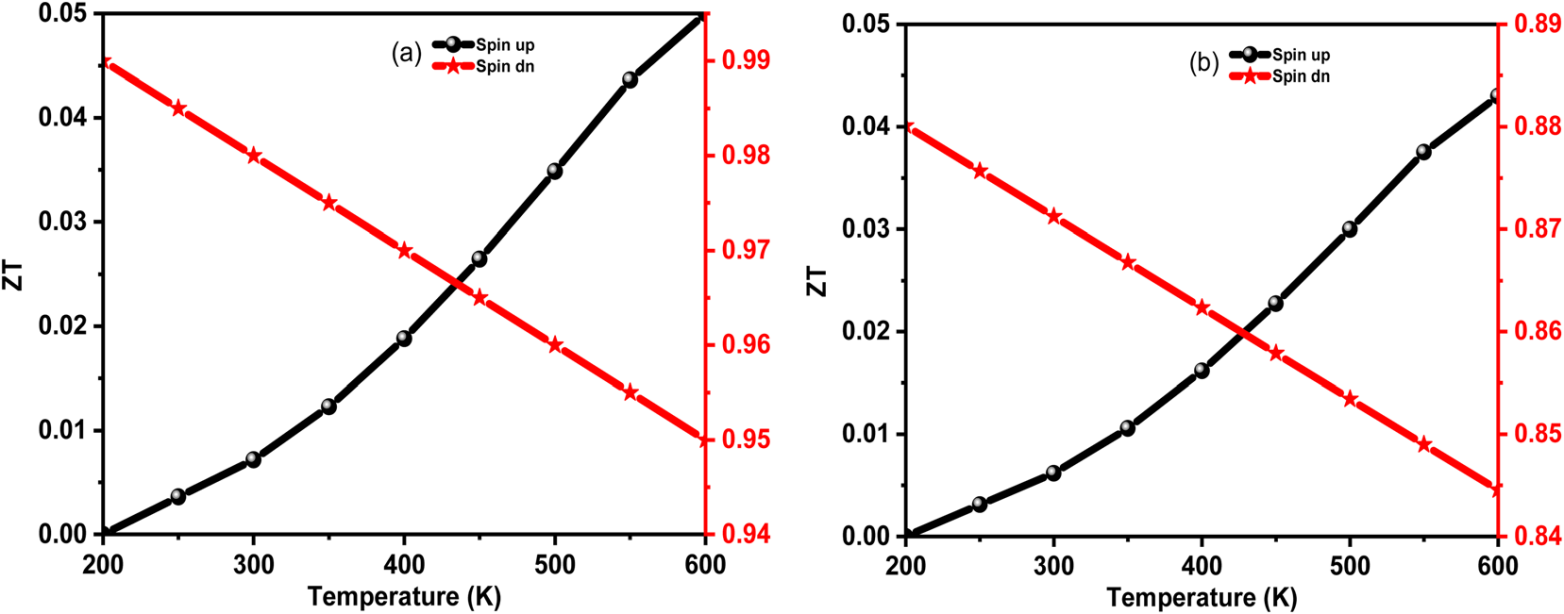
The calculated *ZT* value of (a) K_2_WCl_6_ and (b) K_2_WBr_6_ as a function of temperature.

Motivated by large *S*, *σ*, and PF with low *k*_e_, we have calculated the temperature variation of thermoelectric figure of merit (*ZT*) as Fig. 9(a and b). The detailed analysis revealed that the *ZT* for spin upstate in K_2_WCl_6_ (K_2_WBr_6_) increases from 0.001 (0.001) at 200 K to 0.05 (0.041) at 600 K. On the other hand, *ZT* values of a spin-down channel in K_2_WCl_6_ (K_2_WBr_6_) gradually decrease from 0.99 (0.884) at 200 K to 0.95 (0.845) at 600 K. It is noticeable that spin down channel *ZT* value is dominant in both cases. The higher *ZT* value of the spin-down channel is due to its very low thermal conductivity. Also, the large *ZT* values demonstrated that K_2_W(Cl/Br)_6_ are potential thermoelectric materials.

## 4. Conclusion

To summarize this article, we have systematically investigated the structural, electronic, and thermoelectric properties of K_2_W(Cl/Br)_6_. The negative formation energy leads to the thermodynamic stability of K_2_W(Cl/Br)_6_. In addition, the spin-polarized band structure and density of states calculations revealed the presence of half-metallic character with higher *T*_c_ values, which are 613 K and 597 K for K_2_WCl_6_ and K_2_WBr_6_, respectively. Thus, K_2_W(Cl/Br)_6_ are potential candidates for spintronics application. Furthermore, the origin of half-metallic ferromagnetism is discussed with the double-exchange mechanism. Finally, we have computed the temperature variation of *k*_l_,*σ*, *k*_e_, *S*, PF, and *ZT*. The higher *ZT* values for spin-down channels have resulted from ultra-low *k*_e_, and high PF. In short, K_2_W(Cl/Br)_6_ are potential thermoelectric and spintronic materials.

## Data availability

The datasets produced for current study would be available from Mr Mukaddar Sk on reasonable request.

## Conflicts of interest

The authors have no conflict of interest.

## Supplementary Material
